# miR-150-5p at the Interface of Cancer Biology and Immune Regulation: Dual Roles, Mechanisms, and Therapeutic Opportunities

**DOI:** 10.7150/jca.119966

**Published:** 2026-05-25

**Authors:** Iris Lin, Pardis Shirkani, Cathie Garnis

**Affiliations:** 1 Department of Integrative Oncology, British Columbia Cancer Research Center, Vancouver, BC V5Z1L3, Canada.; 2 Interdisciplinary Oncology Program, University of British Columbia (UBC), Vancouver, BC V5Z1L3, Canada.; 3 Division of Otolaryngology, Department of Surgery, University of British Columbia, Vancouver, BC V5Z1M9, Canada.

## Abstract

MicroRNAs (miRNAs) are small, non-coding RNAs that regulate gene expression post-transcriptionally and play crucial roles in cancer biology and immune function. Among them, miR-150-5p has emerged as a key regulator with complex, context-dependent roles in both tumorigenesis and immune cell differentiation. This review provides a comprehensive synthesis of current knowledge on miR-150-5p, highlighting its dual function as a tumor suppressor or oncogene depending on cancer type and cellular context. We examine its involvement in hematologic malignancies and solid tumors, detailing the molecular mechanisms through which it influences proliferation, apoptosis, and metastasis. Particular emphasis is placed on the role of extracellular vesicle (EV)-associated miR-150-5p as a modulator of the tumor microenvironment (TME), including its impact on angiogenesis, immune evasion, and intercellular communication. We further explore miR-150-5p's regulation of key immune cell subsets—such as macrophages, dendritic cells, T cells, and natural killer cells—and its implications for anti-tumor immunity. Finally, we discuss the therapeutic potential and challenges of targeting miR-150-5p, including delivery barriers, off-target effects, and opportunities for personalized medicine. By integrating recent findings, this review underscores miR-150-5p's value as both a biomarker and a therapeutic target in cancer immunology.

## Introduction

MicroRNAs (miRNAs) are small, non-coding RNA molecules approximately 18-25 nucleotides in length that play a crucial role in post-transcriptional gene regulation. They exert their regulatory function by binding to complementary sequences in the 3' untranslated regions (3' UTRs) of target messenger RNAs (mRNAs), leading to mRNA degradation or translational repression. Since their discovery, miRNAs have been recognized as key regulators of various biological processes, including cell differentiation, proliferation, apoptosis, and immune responses 1,2, while their dysregulation has been implicated in numerous diseases, including cancer, cardiovascular disorders, and autoimmune conditions 3. The effect exerted by miRNAs is in large part realized through intercellular transmission of extracellular vesicles (EVs), which sort and package miRNAs 4. Thus, profiling the expression of miRNAs in EVs may provide insight into disease type, stage, and prognosis 5-7.

In this review, we focus on miRNA-150, which is a key player in normal hematopoiesis, and differentially expressed in hematopoietic cell lineages depending on developmental stage or presence of hematological malignancy 8. miR-150 is also aberrantly expressed in various solid tumors 9-14, suggesting a potential application as a tumor biomarker or therapeutic target. With advancements in EV-derived miRNA detection and isolation techniques and technology, and given the improved sensitivity and specificity of EV-derived miRNA compared to conventional serum biomarkers in the detection of cancer, there is a need to summarize existing literature regarding miR-150, its role in health and disease, and its potential therapeutic applications.

This review provides a comprehensive synthesis of current knowledge on miR-150-5p, highlighting its dual function as a tumor suppressor or oncogene depending on cancer type and cellular context. We examine its involvement in both hematologic malignancies and solid tumors and we discuss the therapeutic potential and challenges of targeting miR-150-5p. By integrating recent findings, this review underscores miR-150-5p's value as both a biomarker and a therapeutic target in cancer immunology.

## miR-150-5p: Biogenesis and Activation

### Biogenesis of miR-150 (Figure 1)

miR-150 is transcribed from the MIR150 gene by RNA polymerase II. The pre-miRNA strands are transported to the cytoplasm where they are cleaved by Dicer into the miRNA duplex 15,16. The guide strand, miR-150-5p, is incorporated into the RNA-induced silencing complex (RISC), which identifies target messages and results in target mRNA cleavage or repression of translation 15.

### Specific Pathways Regulating miR-150-5p Expression

The activation of miR-150 is regulated at both transcriptional and post-transcriptional levels through various pathways, depending on the cell-type and context. In homeostasis, miR-150 plays crucial roles in hematopoietic and immune cell differentiation, and is found accumulated in the lymph nodes, spleen, and thymus 17. Studies have found that miR-150 is highly expressed in mature B- and T-cells, but not their progenitors 3,18. The processes mediating this expression in B-cells involves the transcription factor c-MYB, which contains two miR-150 binding sites in the 3'UTR of the mRNA 18. In the T-cell lineage, miR-150 is increasingly expressed during the maturation process 19, being highly expressed in mature T-cells, but expressed at low levels in thymocytes 19. The mechanism of this pattern is not well studied, but some have found that the Notch signalling pathway plays a role 19. This pathway is essential in controlling cell cycle progression and apoptosis, as well as T-cell progenitor differentiation 19. In particular, miR-150 appears to drive maturation of T-cell progenitors through suppression of NOTCH3 19. In addition to its role in hematopoiesis, miR-150 has been found in the plasma and in various tumor tissues, where it may act as an oncogene or tumor suppressor.

## Cancer Type-Specific Roles of miR-150-5p

miRNA-150-5p has emerged as a key modulator in cancer biology, demonstrating diverse and context-specific roles that underscore its complexity. In some cancers, such as pancreatic, ovarian, and head and neck squamous cell carcinoma, miR-150 is down-regulated, acting as a tumor suppressor gene 20-22. However, overexpression of miR-150 has also been documented in other cancers, such as breast cancer 9. Its dysregulation significantly influences tumor biology through direct modulation of tumor growth, apoptosis, and metastasis, while simultaneously shaping the tumor immune microenvironment 17,23. Table 1 summarizes the role of miR-150 in various cancers.

### Hematological Malignancies: Leukemias and Lymphomas

MiR-150-5p plays a pivotal role in normal hematopoiesis, critically influencing blood cell differentiation. In hematological malignancies, such as in acute myeloid leukemia (AML) and chronic myeloid leukemia (CML), miR-150-5p acts primarily as a tumor suppressor whose loss significantly contributes to oncogenesis 24.

One key target of miR-150-5p is the MYB transcription factor, known to promote proliferation while inhibiting differentiation in hematopoietic stem/progenitor cells. Normally, miR-150 maintains hematopoietic homeostasis by modulating MYB expression. However, its silencing—frequently due to DNA methyltransferase 1 (DNMT1)-driven promoter hypermethylation—leads to unregulated MYB activity, causing continuous proliferation and impaired differentiation 24. Restoration of miR-150-5p in leukemia models reinstates differentiation and arrests growth, underscoring its tumor-suppressive function 24.

MiR-150-5p additionally regulates other oncogenic factors, including the receptor tyrosine kinase FLT3 and the anti-apoptotic protein Survivin (BIRC5). Reduced miR-150 expression facilitates the upregulation of these oncogenic proteins, thereby enhancing the survival and self-renewal capacity of malignant hematopoietic cells 17,24,25.

In B-cell lymphomas, such as diffuse large B-cell lymphoma (DLBCL), miR-150-5p suppression also contributes significantly to malignancy. Patients with DLBCL frequently exhibit diminished miR-150-5p levels, leading to increased FOXP1 expression. FOXP1, an oncogenic transcription factor, amplifies B-cell receptor signaling and Nuclear Factor kappa-B (NF-κB) activation, promoting lymphoma proliferation and survival while simultaneously repressing pro-apoptotic genes such as BIK and HRK 17. Clinically, low miR-150-5p expression correlates with poorer outcomes, observed across other B-cell malignancies including mantle cell lymphoma (MCL) and Burkitt lymphoma, largely due to dysregulated targets like Survivin 17.

Interestingly, not all hematological malignancies display miR-150-5p suppression. In chronic lymphocytic leukemia (CLL) and myelodysplastic syndromes (MDS), paradoxically elevated levels of miR-150-5p have been noted, possibly due to distinct regulatory mechanisms or subtype-specific pathways. Nonetheless, more aggressive forms of CLL typically exhibit miR-150-5p loss, reinforcing its predominantly tumor-suppressive identity 17.

In summary, miR-150-5p functions primarily as a critical tumor suppressor in hematologic cancers. Its downregulation disrupts the suppression of key oncogenic factors, such as MYB, FOXP1, and Survivin, facilitating leukemogenesis and lymphomagenesis. These findings position miR-150-5p as a valuable diagnostic and prognostic biomarker and highlight its therapeutic potential in blood cancers.

### Solid Tumors

miR-150-5p exhibits context-dependent roles in solid tumors, functioning variably as an oncogene or tumor suppressor based on tumor type and molecular environment.

In breast cancer, miR-150-5p primarily serves as an oncogenic miRNA (oncomiR). Elevated miR-150-5p expression correlates with increased tumorigenicity, promoting proliferation and inhibiting apoptosis. Specifically, Huang *et al*. demonstrated that miR-150-5p targets the pro-apoptotic P2X7 receptor, allowing tumor cells to evade apoptosis and enhancing their clonogenicity and invasiveness 9. However, in triple-negative breast cancer (TNBC), miR-150-5p levels are significantly reduced, indicating potential tumor-suppressive activity, highlighting the complexity and subtype-specific roles of miR-150-5p 23.

Similarly, in non-small cell lung cancer (NSCLC), miR-150-5p functions predominantly as an oncomiR, promoting tumor progression. High expression levels of miR-150-5p correlate with poor prognosis and increased metastatic potential 26-28. Mechanistically, miR-150-5p targets critical tumor suppressors, including Forkhead box protein O4 (FOXO4), which normally induces apoptosis and cell-cycle arrest. By suppressing FOXO4, miR-150-5p activates pro-metastatic pathways such as AKT/PI3K signaling and epithelial-mesenchymal transition (EMT), enhancing lung cancer metastasis 26. The Sirtuin 2 (SIRT2)/JMJD2A pathway is also implicated in the disease, where high miR-150 expression suppresses SIRT2 expression, a negative regulator of JMJD2A, ultimately leading to enhancement of cellular proliferation, migration, and invasion 27.

In gastric carcinoma, miR-150-5p acts as an oncogenic factor, frequently upregulated in tissues and serum of affected patients. miR-150-5p promotes gastric cancer progression by directly suppressing the pro-apoptotic transcription factor Early Growth Response 2 (EGR2), enhancing proliferation and cell survival 13. Downregulation of miR-150-5p reverses these effects, affirming its oncogenic role. Furthermore, miR-150-5p influences other critical pathways, including AKT/PI3K signaling, EMT, and potentially p53-regulated genes associated with metastasis and invasion 23,28,29.

miR-150-5p demonstrates diverse roles in other cancers, showing tumor-suppressive activity in hepatic, esophageal, and colorectal malignancies through targets such as GRB2-associated binding protein 1 (GAB1), Zinc Finger E-Box Binding Homeobox 1 (ZEB1), and Vascular Endothelial Growth Factor A (VEGFA), respectively 30-32. Conversely, miR-150-5p acts predominantly as an oncogene in cervical cancers, promoting tumor progression through targeting of the PDCD4 tumor suppressor gene 23,33. Common molecular pathways modulated by miR-150-5p across various cancers include NF-κB signaling, Wnt/β-catenin pathways, EMT regulators such as High Mobility Group AT-Hook 2 (HMGA2), and matrix metalloproteinases (MMP) like MMP-13/14 23.

Overall, the role of miR-150-5p in solid tumors varies significantly depending on cancer type and cellular context. This dual functionality highlights its potential both as a therapeutic target and as a diagnostic or prognostic biomarker, warranting further investigation to guide tailored therapeutic strategies.

## Extracellular Vesicle Associated miR-150-5p in Cancer

### EV-Mediated Modulation of the Tumor Microenvironment

EVs are small, membrane-bound particles secreted by virtually all cell types, including cancer cells, serving as critical mediators of intercellular communication 36. Tumor-derived EVs encapsulate diverse bioactive molecules such as proteins, lipids, DNA, and RNA, notably miRNAs, enabling their protected transport and targeted delivery to recipient cells within the tumor microenvironment (TME) 36. By transferring these molecular cargos, tumor-derived EVs significantly influence key biological processes including immune modulation, cell proliferation, migration, angiogenesis, and metastasis 37. Consequently, they play a pivotal role in cancer progression, immune evasion, and shaping the complex dynamics of the TME, dictated by the nature of their cargo 37.

miRNA loading into EVs is not random but highly regulated: the miRNA profiles of EVs are markedly different from those of their parent cells 36, and only a minor subset of EVs carries most of the miRNA cargo, indicating heterogeneous miRNA distribution among vesicles 36. In the case of miR-150-5p, this miRNA has been found in EVs from multiple cell types. For example, neutrophil-derived EVs (from immune cells) contain miR-150-5p 38. Likewise, hypoxia-treated kidney tubular epithelial cells secrete EVs highly enriched in miR-150-5p 39. These findings show that miR-150-5p is secreted via EVs from both normal (e.g. immune or stem) cells and tumor cells. Whether miR-150-5p is disproportionately enriched in EVs versus cellular levels is not fully established, as quantitative comparisons are limited.

Among these examples, one of the most striking involves lung cancer under hypoxic stress**.** Hypoxia drives lung tumor cells to release EVs heavily loaded with miR-150-5p, which are taken up by natural killer (NK) cells 40. The transferred miR-150-5p targets *CD226* (an activating NK cell receptor), resulting in NK cells with diminished cytotoxicity and reprogrammed phenotypes 40. NK cells exposed to these tumor-derived EVs exhibit lower surface CD226 and reduced interferon-γ (IFN- γ) production, along with a paradoxical increase in pro-angiogenic factors like VEGF and MMPs that support tumor growth 40. This EV-mediated crosstalk effectively blunts immune surveillance and favors tumor progression in the TME.

On the other hand, EV transfer of miR-150-5p is not unidirectional - immune cells can also secrete miR-150-5p in vesicles to their surrounding environment. Notably, M1-polarized macrophages release exosomes enriched in miR-150-5p that can enter glioma cells and suppress their invasiveness 41. The internalized miR-150-5p in tumor cells directly downregulates *MMP-16*, curbing matrix degradation and tumor motility, and thereby inhibits glioma progression 39. These findings illustrate that EV-associated miR-150-5p serves as a key communicator in the TME, capable of both dampening anti-tumor immunity (when delivered from tumor to immune cells) and enhancing anti-tumor effects (when delivered from immune cells to tumor). In addition to immune cells, other stromal components are likely involved: for instance, cancer-associated fibroblasts have been shown to shuttle the passenger strand miR-150-3p in exosomes to tumor cells, with loss of that anti-tumoral signal accelerating tumor growth 42.

By extension, EV-carried miR-150-5p may also partake in fibroblast-tumor or endothelial-tumor communication, although direct evidence is still emerging. Taken together, extracellular vesicles serve as vehicles for miR-150-5p-mediated crosstalk, with significant consequences for tumor angiogenesis, metastasis, and the balance of immune attack versus evasion in the microenvironment 42.

### EV-associated miR-150-5p as a Prognostic Biomarker

Increasing evidence indicates that miR-150-5p packaged in EVs can serve as a circulating biomarker of cancer prognosis. In colorectal cancer, for example, patients with low serum exosomal miR-150-5p levels tend to have more aggressive disease and shorter survival, whereas higher exosomal miR-150-5p is associated with better outcomes 43. Similar trends are observed in other malignancies: in prostate cancer, EV-derived miR-150-5p is significantly under-expressed in patients with high Gleason scores and metastatic disease 44. Pathway analyses suggest that EV-miR-150-5p may normally restrain oncogenic pathways (e.g. Wnt signaling) involved in bone metastasis 44. Likewise, in breast cancer, reduced levels of miR-150-5p in serum EVs correlate with advanced tumor grade and larger tumor size 45. These clinical studies from the past few years consistently point to exosomal miR-150-5p as a favourable prognostic factor in multiple cancers, with low EV-miR-150-5p often marking a higher risk of progression or poorer survival 43,44. EV-miR-150-5p thus shows promise as a minimally invasive liquid biopsy marker for patient stratification. However, standardization of detection methods and normalization strategies remains lacking. There is a critical need for robust, reproducible assays and reference standards to facilitate clinical translation and comparison across studies. Future research must address methodological discrepancies and validate EV-miR-150-5p across diverse patient cohorts and cancer types. Further, research efforts to understand the bi-directional effect of miR-150 within the TME may aid in validation and discovery of diagnostic biomarkers.

## The Role of miR-150-5p in Immune Cell Development and Function

miR-150-5p plays critical regulatory roles in immune cell biology, influencing both innate and adaptive immunity through its precise modulation of immune cell development, differentiation, and functional responses 17 (Table 2). By targeting key transcription factors and signaling pathways, miR-150-5p orchestrates complex immune processes that shape the anti-tumor immune response, directly impacting macrophages, dendritic cells, NK cells, and lymphocyte populations 17,46-48. Its ability to fine-tune immune cell phenotypes underscores its dual and context-dependent activities, which can promote either immunological tolerance or enhanced immune surveillance against cancer. Understanding the mechanisms underlying miR-150-5p-mediated immune regulation provides valuable insights into cancer immunology and opens new avenues for therapeutic interventions aimed at harnessing or modulating the immune system.

### Macrophages and Tumor-Associated Macrophages

Macrophages are key innate immune cells whose phenotypic plasticity allows them to polarize into pro-inflammatory (M1) or anti-inflammatory (M2) states 49. In the tumor microenvironment, tumor-associated macrophages (TAMs) typically exhibit an M2-like phenotype, contributing to angiogenesis, immunosuppression, and tumor progression. miR-150-5p has emerged as a critical modulator of TAM function 47.

Zhang *et al*. demonstrated that tumor-derived micro-vesicles enriched with miR-150 are internalized by TAMs, leading to increased secretion of VEGF, thereby promoting tumor angiogenesis and growth. Inhibiting miR-150 *in vivo* reduced VEGF levels and suppressed tumor progression, highlighting a pro-tumoral role for miR-150-5p in this context 50.

Conversely, recent studies have shown miR-150-5p can also support anti-tumoral macrophage activity. In glioma, for example, Yan *et al*. reported that exosomes derived from M1 macrophages deliver miR-150-5p to glioma cells, where it downregulates MMP-16 and inhibits tumor invasion and growth 41. Similarly, in colorectal cancer, Cao *et al*. showed that circFMN2 functions as a sponge for miR-150-5p in TAMs. Its knockdown enhanced miR-150-5p activity, promoted M1 polarization, and inhibited tumor-supportive M2 markers, such as CD163 and CCL22 51.

These findings highlight the context-dependent nature of miR-150-5p in macrophages—acting as either a tumor promoter or suppressor depending on its source, targets, and delivery mechanism.

### Dendritic Cells

Dendritic cells (DCs) play a critical role in bridging innate and adaptive immunity by presenting tumor antigens to T cells. miR-150-5p has been shown to regulate the maturation and cytokine profile of DCs 46. For instance, in a model of sterile inflammation, necrotic cell debris provoked DC activation accompanied by *down*-regulation of miR-150, whereas experimental overexpression of miR-150 in DCs dampened their response 52. Zhu *et al*. found that elevating miR-150 levels attenuated the upregulation of DC maturation markers (CD40, CD86) and reduced pro-inflammatory cytokine secretion via suppression of the JAK1/STAT1 and AP-1 (c-Fos) signaling pathways 52. These data suggest that miR-150-5p acts as a negative regulator of DC activation. In the context of cancer, such an effect could translate to reduced DC-mediated priming of anti-tumor T cells if miR-150-5p is highly expressed in DCs. Conversely, low miR-150-5p in DCs (as might occur in an inflammatory tumor milieu) would permit stronger DC activation and possibly better anti-tumor immune responses. While direct evidence in tumors is still emerging, the known ability of tumors to induce tolerogenic or immature DCs raises the possibility that dysregulation of miR-150-5p in intratumoral DCs could contribute to immune evasion.

### Natural Killer Cells

NK cells are innate lymphocytes critical for direct tumor cell killing and cytokine production. MiR-150-5p plays a pivotal role in NK cell development and functional maturation. It is highly expressed during NK cell ontogeny, and enforced miR-150 expression in mice accelerates the generation of mature NK cells 53. *In vivo* loss-of-function studies demonstrated that miR-150 is required for NK lineage development: mice genetically deficient in miR-150 exhibit an intrinsic defect in producing mature NK cells 53. Mechanistically, miR-150 promotes NK cell differentiation at least in part by targeting the transcription factor c-Myb, which is a negative regulator of NK maturation 47. Thus, miR-150-5p is essential for the proper formation of the NK cell compartment, which is a first line of defence against tumors.

Paradoxically, while miR-150-5p fosters NK cell development, it can restrain certain effector functions in *mature* NK cells. High miR-150 levels have been associated with reduced NK cell cytotoxicity. Notably, miR-150 directly targets the 3′UTR of PRF1 (perforin), the gene encoding the perforin protein that NK cells use to kill target cells 47. By downregulating perforin expression, miR-150 can diminish the lytic capacity of human NK cells 47.

MiR-150 (along with miR-27a, miR-378, and others) also contributes to a microRNA program that suppresses the cytotoxicity of the CD56^dim NK subset, which is the highly cytotoxic subset in humans 47. In mice, miR-150 and a set of other miRNAs were shown to limit NK cell production of interferon-γ during activation 47, further indicating a role in tuning the activation threshold of NK cells.

Additionally, miR-150 can promote apoptotic pathways in NK cells by upregulating pro-apoptotic factors like Bim and p53 47. This suggests that excessive miR-150-5p might reduce NK cell survival or persistence.

In summary, miR-150-5p has a dual role in NK biology: it is indispensable for NK cell differentiation and maturation yet acts as a negative regulator of NK cell activation and cytotoxic function when highly expressed 53. The net effect of miR-150-5p in NK cells is, therefore, stage-specific - promoting the development of the NK repertoire but restraining NK anti-tumor activity if not properly downregulated at the effector stage.

### B-Lymphocytes

B-lymphocytes, or B-cells, are an integral part of adaptive immunity, responsible for humoural immunity through the production of antibodies 54. Progenitor B-cells (pro-B) differentiate into precursor cells (pre-B) and immature B-cells expressing membrane-bound immunoglobulin M (IgM) through a gradual process in the bone marrow consisting of transitional stages 55. The immature B-cells then exit the bone marrow to undergo additional differentiation in the secondary lymphoid organs, resulting in a progeny of B-cells with various receptors 56. Some B-cells will further differentiate into plasma cells, while some remain in the secondary lymphoid organ, and others differentiate into memory B-cells 56.

miR-150 serves as a critical regulator throughout this process of B-lymphocyte maturation 17; Zhou *et al*. have found that the ectopic expression of miR-150 inhibits pro-B cell differentiation into pre-B cells, and the formation of mature B-cells 57. This may be due in part to the effect of miR-150 on the c-Myb pathway. During early B-cell development in the bone marrow, miR-150 expression is actively suppressed to permit c-Myb-driven proliferation of pro-B cells 18. As B cells mature, miR-150 expression increases dramatically, creating a negative feedback loop that downregulates c-Myb and prevents excessive proliferation 17. This regulatory mechanism ensures proper transition through developmental checkpoints.

Researchers have found that genetic ablation of miR-150 resulted in expansion of mature B-cell subsets 18, and significantly higher levels of immunoglobulins in the blood 58, showing that miR-150 also affects antibody production 18. Additionally, miR-150 directly targets FLT3, resulting in a reduction of B-cell proliferation and decreased IgM expression on the cell surface 59.

The BCR pathway is also implicated; Kluiver *et al*. found that high levels of miR-150 during the transitional phases of B-cell maturation results in BCR-mediated apoptosis of transitional B-cells and suppression of B-cell growth 60. On the other hand, lowered miR-150 expression in transitional B-cells inhibited BCR-mediated apoptosis and promoted the overproduction of self-autoreactive B-cells 60.

### T-Lymphocytes

Another crucial component of the adaptive immune response involves the T-lymphocyte, or T-cell, response, which facilitates cell-mediated immunity 37. The process of T-cell development begins as haematopoietic stem cells (HSC) in the fetal liver and later in the bone marrow differentiate into multipotent progenitors. Some of these cells then migrate to the thymus where they eventually develop into mature and functional T-cells 56.

The importance of miRNAs in T-cell development has been illuminated through studies showing that T-cell development is hindered in the absence of Dicer 61. If Dicer is deleted after selection in the thymus, effector T-cells are produced, but are not able to mount a cytotoxic response to an infection 62. microRNA sequencing of various T-cell subsets found that miR-150 was the most highly expressed miRNA in naïve T-cells, central memory T-cells, effector T-cells, and cytotoxic CD8+ T-cells, but expressed at a low level in stem cell-like memory T-cells (which are the earliest subset after cells enter progressive differentiation following T-cell activation), suggesting that this specific miRNA is key in T-cell differentiation 48,63. Target gene analysis showed that FOXP1 and RC3H1 were key targets of miR-150, and result in T-cell differentiation, aggregation and activation 63. However, overexpression of miR-150 In T-cells resulted in increased apoptosis and inhibition of T-cell proliferation 63. Further studies have shown that miR-150 impedes memory formation in CD8+ T-cells 64,65.

The impact of miR-150 on T-cell function is also multifaceted. miR-150 expression increased cytokine secretion, meaning that effector T-cell function was enhanced 63. Smith *et al*. found that miR-150 knockdown in CD8+ T-cells resulted in poor expansion and differentiation into short-lived terminal cytotoxic cells that mount a weaker response after transitioning into memory T-cells 48. However, Ménoret *et al*. demonstrated that miR-150 regulated IL-2 secretion and apoptosis in CD4+ T-cells, hindering their ability to help CD8+ T-cells 66.

## Therapeutic Potential of miR-150-5p

### Specificity and Off-Target Effects

Targeting miR-150-5p for therapy necessitates careful consideration of specificity and off-target effects. By nature, miRNAs have numerous mRNA targets - miR-150-5p can potentially bind hundreds of transcripts - so introducing a miR-150 mimic or inhibitor could unintentionally dysregulate genes beyond the intended targets.

Ensuring that a miR-150-5p-based therapy affects only the desired pathways (for example, those driving tumor immune evasion) without perturbing essential functions in normal tissues is a major challenge. One concern is that miR-150-5p plays normal roles in the immune system (e.g. in B, T, and NK cell differentiation; a systemic therapy might disrupt immune homeostasis).

Indeed, a **“**double-edged sword**”** nature of miR-150 has been noted, where it can be tumor-suppressive in some cells but oncogenic in others 23. This contextual duality means an intervention could have opposing effects depending on cell type.

Off-target gene repression is also a significant safety issue. A miRNA mimic could silence partial complement sequences in unintended genes, while an anti-miR could bind other RNAs. Early-generation miRNA therapies revealed such risks: for example, MRX34 (a miR-34a mimic) - although targeting a tumor-suppressive miRNA - caused serious immune-related adverse events in patients, partly due to off-target immune activation 67. All 47 patients in the MRX34 trial experienced immune toxicities (grade III inflammatory reactions in some), leading to trial termination 67.

These outcomes underscore that immune off-target effects (such as unintended activation of innate immunity by the RNA payload or nanoparticle) are a real hazard for miRNA therapeutics. Thus, achieving high specificity for miR-150-5p will likely require advanced designs - for instance, chemically modifying oligonucleotides (locked nucleic acids, etc.) to enhance target selectivity, and perhaps incorporating tissue-specific promoters or targeting moieties so that the miR-150 therapy is active only in the tumor or immune compartment of interest. Ongoing research is focused on minimizing these off-target interactions (e.g. by improving the miRNA seed specificity) to make miR-150-5p therapies safer 68,69.

### Delivery Barriers

Effective delivery of miRNA-based drugs into tumors remains a formidable barrier. Naked miRNA mimics or inhibitors are rapidly degraded by RNases in the bloodstream and can be cleared by the renal and reticuloendothelial systems before reaching the tumor. Moreover, even if they survive circulation, getting these molecules across the vascular endothelium, through the tumor stroma, and into the cytoplasm of target cells is a significant hurdle.

A range of delivery strategies is being explored to overcome these hurdles 67. Local delivery, such as direct intratumoral injection, can achieve high concentrations but is not feasible for all tumors (especially metastatic or internal lesions). Systemic delivery vectors include viral vectors, lipid nanoparticles, polymeric nanoparticles, and even engineered exosomes as natural carriers 67.

Each method has its challenges: viral vectors can have immunogenicity and insertional mutagenesis concerns, while nanoparticles must be optimized for stability and tumor uptake. One key requirement is to protect the miR-150 mimic/inhibitor from degradation en route - liposomal encapsulation (as with MRX34) and chemical modifications (2'-O-methyl, phosphorothioate backbones, etc.) have been used to improve stability 67.

Another issue is specific tumor targeting: nanoparticles can be functionalized with ligands (e.g. antibodies, peptides) to preferentially bind receptors on tumor cells, delivering miR-150 modulators to the tumor site while sparing normal tissues 67. Even with targeting, however, only a fraction of the injected dose may accumulate in the tumor. Additionally, the dense extracellular matrix in some tumors and high interstitial pressure can impede deep penetration of these molecules.

An often-overlooked barrier is endosomal escape. Once a miRNA mimic or inhibitor enters a cell via endocytosis (common for nanoparticle uptake), it may be trapped in endosomes and degraded unless an efficient release mechanism is in place. Formulations are now incorporating endosomolytic peptides or pH-responsive polymers to facilitate cytosolic release of miRNA cargo.

Finally, immune recognition of the delivery system poses a challenge: the body's innate immune sensors might detect double-stranded RNA mimics or the delivery particles themselves as foreign. Infusion reactions (fever, chills) observed in miRNA therapy trials (e.g. patients receiving the miR-16 mimic “TargomiRs” often experienced fever and immune activation 67) highlight the need to cloak or refine these delivery vehicles to avoid clearance by phagocytes and unintended immune stimulation.

### Opportunities for Personalized Medicine and Combination Strategies

Despite the challenges, there are exciting opportunities to harness miR-150-5p in personalized cancer therapy. Given its context-dependent role, a tailored approach is essential - one patient's tumor might have downregulated miR-150-5p to escape immune attack, whereas another's tumor might exploit high miR-150-5p to suppress immunity. Profiling miR-150-5p levels (and its downstream gene targets) in individual tumors could guide therapeutic strategy.

For patients whose tumors exhibit *low* miR-150-5p along with poor immune cell infiltration, a miR-150-5p mimic therapy might be beneficial to restore this miRNA's tumor-suppressive, immune-recruiting function. Conversely, in tumors (like certain lung cancers) where miR-150-5p is aberrantly *high* and linked to immune evasion (e.g. NK cell dysfunction), using a miR-150-5p inhibitor (antagomiR) could relieve that suppression. This personalized deployment maximizes efficacy while minimizing the risk of pushing a tumor's biology in the wrong direction.

Notably, miR-150-5p could also be integrated as a biomarker for immunotherapy. High endogenous miR-150 in a tumor might predict a more inflamed TME and better responsiveness to checkpoint inhibitors, whereas cases with low miR-150 and an immune-cold phenotype might benefit from miR-150 mimic “priming” before or alongside immunotherapy.

From a therapeutic development standpoint, combining miR-150-5p modulation with existing treatments offers synergistic possibilities. For example, adding a miR-150-5p mimic to immune checkpoint blockade might convert an immunologically “cold” tumor into a “hot” one, rendering PD-1/PD-L1 antibodies more effective.

On the other hand, combining a miR-150 inhibitor with conventional therapies could be useful in scenarios like the hypoxic lung cancer model. One could envision using an anti-miR-150 alongside NK cell adoptive transfer or IL-15 therapy to ensure the introduced NK cells maintain their activity in the TME. Early preclinical evidence supports combination benefits: in a mouse lung cancer model, systemic delivery of a miR-150-5p inhibitor significantly enhanced tumor control and reinstated NK cell cytotoxic function 40, hinting that anti-miR-150 therapy could work in concert with the immune system's natural fighters.

Integrating miR-150-5p into personalized medicine extends beyond traditional drug therapies and encompasses innovative approaches such as vaccine development and cellular therapies. *Ex vivo* modification strategies could involve engineering patient-derived T cells or NK cells with altered miR-150-5p expression. For instance, knocking down miR-150 in NK cells in cases where tumors secrete miR-150-rich exosomes, before cellular infusion. Additionally, targeting downstream genes regulated by miR-150-5p, such as c-Myb or MMP16, with small-molecule inhibitors may achieve similar therapeutic outcomes by indirectly modulating miR-150-5p activity. Thus, miR-150-5p provides a strategic basis for personalized immunotherapy, where therapeutic regimens are tailored based on individual tumor miR-150-5p expression profiles.

Future clinical trials must incorporate stratification by tumor miR-150 expression and immune characterization to optimize therapeutic efficacy. Personalized interventions, guided by molecular diagnostics, could significantly enhance the therapeutic index and facilitate precise, effective utilization.

## Conclusion

In conclusion, miR-150-5p serves as a critical regulatory nexus between cancer progression and immune responses, influencing tumor cell biology and immune cell functionality within the tumor microenvironment. Its dual role as both tumor promoter and suppressor underscores the complexity of its biological actions, which are highly context-dependent. Clinically, miR-150-5p holds considerable promise as both a prognostic biomarker and a therapeutic target. Modulation of miR-150-5p has demonstrated significant antitumor effects in preclinical models, either by restoring immune cell functionality or by sensitizing tumors to existing immunotherapies. Future research focused on improving targeted delivery methods, validating therapeutic strategies across diverse cancer models, and integrating miR-150-5p-based treatments into combination regimens will be crucial to translating these findings into clinical applications. Ultimately, miR-150-5p represents a promising target in cancer immunotherapy, offering novel avenues to enhance treatment efficacy and improve patient outcomes.

## Figures and Tables

**Figure 1:**
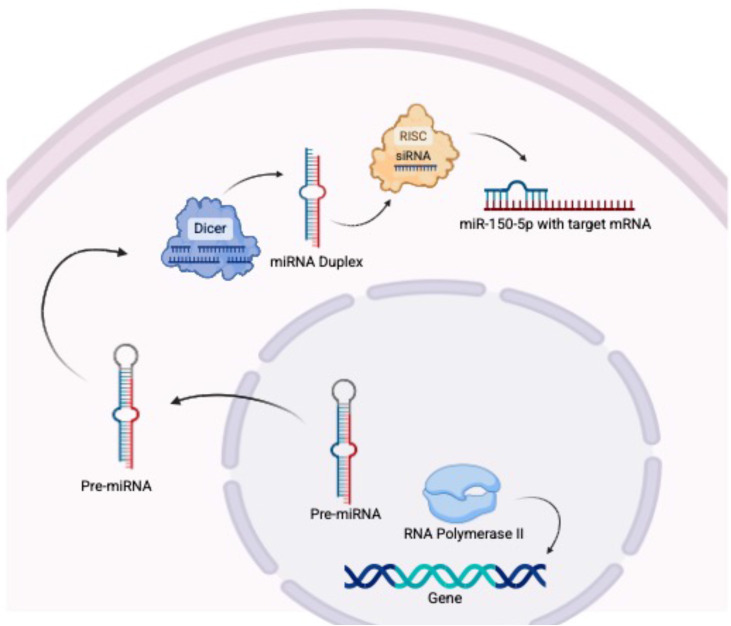
In the biogenesis of miR-150, RNA polymerase II transcribes miR-150 from the MIR150 gene, before the pre-miRNA strands (miR-150-3p and miR-150-5p) are transported to the cytoplasm where they are cleaved by Dicer into a double-stranded miRNA duplex. The guide strand, miR-150-5p, is incorporated into the RNA-induced silencing complex (RISC), while miR-150-3p is degraded. Mature miR-150-5p then binds to its target mRNA.

**Table 1:** Dual roles of miR-150-5p in hematological malignancies and solid tumors.

Cancer Type	Expression	Role	Key Targets	Mechanisms and Effects	Clinical Implications	References
Hematological Malignancies						
Acute Myeloid Leukemia	↓	Tumor suppressor	MYB, FLT3, Survivin	Promoter hypermethylation silences miR-150 → MYB-driven proliferation & impaired differentiation. Restoring miR-150 induces differentiation.	Poor prognosis; potential therapeutic target.	Hu *et al*. 2019 (24)
Chronic Myeloid Leukemia	↓	Tumor suppressor	MYB	DNMT1-mediated hypermethylation → MYB overexpression → leukemogenesis.	Diagnostic biomarker for disease progression.	Hu *et al*. 2019 (24)
Diffuse Large B-Cell Lymphoma	↓	Tumor suppressor	FOXP1	Low miR-150 → FOXP1 upregulation → enhanced NF-κB signaling & anti-apoptotic effects.	Correlates with aggressive disease & poor survival.	Hu *et al*. 2023 (17)
Burkitt Lymphoma	↓	Tumor suppressor	Survivin, c-MYB	High C-MYC → downregulation of miR-150 expression → anti-apoptotic effect. Re-introduction of miR-150 → reduced cell proliferation and apoptosis.	Diagnostic biomarker and potential therapeutic target.	Hu *et al*. 2023 (17) Chen *et al*. 2013 (34)12/06/2025 12:49:00
Chronic Lymphocytic Leukemia	↓ or ↑ (subtype-dependent)	Oncogenic	GAB1, FOXP1	Low levels of miR-150 → increased GAB1 and FOXP2 → increased BCR signaling, leading to increased invasiveness.	Subtype-specific prognostic utility.	Hu *et al*. 2023 (17) Stamatopoulos *et al*. 2015 (35)
Solid Tumors						
Breast Cancer	↑ (non-TNBC)	Oncogenic	P2X7	Inhibits apoptosis via P2X7 suppression; promotes EMT & chemoresistance.	High levels correlate with poor outcomes.	Huang *et al*. 2013 (32)
Non-Small Cell Lung Cancer	↑	Oncogenic	FOXO4, SIRT2, JMJD2A	miR-150 expression → FOXO4 suppression → AKT/PI3K activation → metastasis & therapy resistance. miR-150 expression → SIRT2 suppression → JMJD2A activation → cellular proliferation	Poor prognosis; target for anti-metastatic therapy.	Jiang *et al*. 2018 (9) Li *et al*. 2016 (27)
Gastric Cancer	↑	Oncogenic	EGR2	miR-150 overexpression → EGR2 inhibition → enhanced proliferation & survival.	Potential therapeutic.	Wu *et al*. 2010 (26)
Hepatocellular Carcinoma	↓	Tumor suppressor	GAB1	Low miR-150 expression → increased GAB1 expression → ERK activation → EMT and metastasis.	Potential therapeutic agent.	Sun *et al*. 2016 (30)
Esophageal squamous cell carcinoma	↓	Tumor suppressor	ZEB1	miR-150 targets ZEB1, leading to suppression of E-cadherin and decreased migration and tumorigenicity of ESCC cells.	Potential therapeutic.	Yokobori *et al*. 2012 (31)
Colorectal Cancer	↓	Tumor suppressor	VEGFA	Low miR-150-5p → overexpression of VEGFA → activation of Akt/mTOR pathway → angiogenesis, proliferation, survival, metastasis.	Potential diagnostic biomarker and therapeutic agent.	Chen *et al*. 2018 (32)
Cervical Cancer	↑	Oncogenic	PDCD4	miR-150 targets PDCD4 → activation of NF-kB, MMP-9, and AKT → tumor cell proliferation, migration and invasion	Potential target for prediction and treatment.	Zhang *et al*. 2018 (33)

**Table 2:** The role of miR-150-5p in immune cell regulation and tumor immunity.

Immune Cell Type	Expression/Activity	Role	Key Targets	Functional Outcome	Implications	References
Innate Immunity						
Macrophages/TAMs	Context-dependent	Pro-tumoral: Enhances M2 polarization. Anti-tumoral: M1-derived exosomes inhibit invasion.	VEGF, MMP16, CD163, CCL22	↑ Angiogenesis (via VEGF); ↓ Invasion (via M1 exosomes).	Target for TAM reprogramming.	Cao *et al*. 2024 (51) Liu *et al*. 2013 (50) Zhou *et al*. 2024 (41) 12/06/2025 12:49:00
Dendritic Cells (DCs)	↓ in inflammation	Suppresses DC maturation and cytokine production.	JAK1/STAT1, AP-1 (c-Fos).	↓ CD40/CD86 expression; ↓ pro-inflammatory cytokines; ↓ T-cell priming.	May contribute to tumor immune evasion.	Scalavino *et al*. 2020 (46) Zhu *et al*. 2017 (52)
Natural Killer (NK) Cells	High during development	Development: Essential for NK maturation. Function: Restricts cytotoxicity.	c-Myb, PRF1 (perforin), IFN-γ	Promotes NK lineage but ↓ perforin/IFN-γ in mature NKs; ↑ apoptosis	Balance needed for anti-tumor NK activity.	Pesce *et al*. 2020 (53) Xu *et al*. 2019 (47)
Adaptive Immunity						
B-Lymphocytes	↑ with maturation	Regulates B-cell maturation and differentiation.	c-Myb, FLT3, BCR	Suppresses early B-cell proliferation; modulates memory vs. plasma cell balance.	Loss linked to B-cell malignancies (e.g., DLBCL).	Xiao *et al*. 2007 (18) Hu *et al*. 2023 (17) Jiang *et al*. 2016 (59) Kluiver & Chen 2012 (60)
T-Lymphocytes	Stage-specific	Development: Promotes T-cell differentiation. Function: Context-dependent.	FOXP1, RC3H1, AKT/mTOR	Drives naive→effector transition; ↓ miR-150 enhances CD4+ survival; ↑ CD8+ memory.	Potential target for adoptive T-cell therapy.	Xia *et al*. 2022 (63) Menoret *et al*. 2023 (66) Smith *et al*. 2015 (48)12/06/2025 12:49:00

## References

[1] Bartel DP (2009). MicroRNAs: target recognition and regulatory functions. Cell.

[2] Ambros V (2004). The functions of animal microRNAs. Nature.

[3] Monticelli S, Ansel KM, Xiao C, Socci ND, Krichevsky AM, Thai TH (2005). MicroRNA profiling of the murine hematopoietic system. Genome Biol.

[4] Xu D, Di K, Fan B, Wu J, Gu X, Sun Y (2022). MicroRNAs in extracellular vesicles: Sorting mechanisms, diagnostic value, isolation, and detection technology. Front Bioeng Biotechnol.

[5] Hu Z, Chen X, Zhao Y, Tian T, Jin G, Shu Y (2010). Serum MicroRNA Signatures Identified in a Genome-Wide Serum MicroRNA Expression Profiling Predict Survival of Non-Small-Cell Lung Cancer. J Clin Oncol.

[6] Dickman CTD, Lawson J, Jabalee J, MacLellan SA, LePard NE, Bennewith KL (2017). Selective extracellular vesicle exclusion of miR-142-3p by oral cancer cells promotes both internal and extracellular malignant phenotypes. Oncotarget.

[7] Lawson J, Dickman C, MacLellan S, Towle R, Jabalee J, Lam S (2017). Selective secretion of microRNAs from lung cancer cells via extracellular vesicles promotes CAMK1D-mediated tube formation in endothelial cells. Oncotarget.

[8] Wang F, Ren X, Zhang X (2015). Role of microRNA-150 in solid tumors (Review). Oncol Lett.

[9] Huang S, Chen Y, Wu W, Ouyang N, Chen J, Li H (2013). miR-150 promotes human breast cancer growth and malignant behavior by targeting the pro-apoptotic purinergic P2X7 receptor. PloS One.

[10] Srivastava SK, Bhardwaj A, Singh S, Arora S, Wang B, Grizzle WE (2011). MicroRNA-150 directly targets MUC4 and suppresses growth and malignant behavior of pancreatic cancer cells. Carcinogenesis.

[11] MiR-150 is associated with poor prognosis in esophageal squamous cell carcinoma via targeting the EMT inducer ZEB1 [Internet] [cited 2025 Apr 15]. Available from: https://onlinelibrary.wiley.com/doi/epdf/10.1111/cas.12030.

[12] Ma Y, Zhang P, Wang F, Zhang H, Yang J, Peng J (2012). miR-150 as a potential biomarker associated with prognosis and therapeutic outcome in colorectal cancer. Gut.

[13] Wu Q, Jin H, Yang Z, Luo G, Lu Y, Li K (2010). MiR-150 promotes gastric cancer proliferation by negatively regulating the pro-apoptotic gene EGR2. Biochem Biophys Res Commun.

[14] Zhang N, Wei X, Xu L (2013). miR-150 promotes the proliferation of lung cancer cells by targeting P53. FEBS Lett.

[15] Okato A, Arai T, Kojima S, Koshizuka K, Osako Y, Idichi T (2017). Dual strands of pre-miR-150 (miR-150-5p and miR-150-3p) act as antitumor miRNAs targeting SPOCK1 in naïve and castration-resistant prostate cancer. Int J Oncol.

[16] Gregory RI, Chendrimada TP, Cooch N, Shiekhattar R (2005). Human RISC couples microRNA biogenesis and posttranscriptional gene silencing. Cell.

[17] Hu YZ, Li Q, Wang PF, Li XP, Hu ZL (2023). Multiple functions and regulatory network of miR-150 in B lymphocyte-related diseases. Front Oncol.

[18] Xiao C, Calado DP, Galler G, Thai TH, Patterson HC, Wang J (2007). MiR-150 Controls B Cell Differentiation by Targeting the Transcription Factor c-Myb. Cell.

[19] Ghisi M, Corradin A, Basso K, Frasson C, Serafin V, Mukherjee S (2011). Modulation of microRNA expression in human T-cell development: targeting of NOTCH3 by miR-150. Blood.

[20] Kim TH, Jeong JY, Park JY, Kim SW, Heo JH, Kang H (2017). miR-150 enhances apoptotic and anti-tumor effects of paclitaxel in paclitaxel-resistant ovarian cancer cells by targeting Notch3. Oncotarget.

[21] Yang K, He M, Cai Z, Ni C, Deng J, Ta N (2015). A decrease in miR-150 regulates the malignancy of pancreatic cancer by targeting c-Myb and MUC4. Pancreas.

[22] Koshizuka K, Nohata N, Hanazawa T, Kikkawa N, Arai T, Okato A (2017). Deep sequencing-based microRNA expression signatures in head and neck squamous cell carcinoma: dual strands of pre- miR -150 as antitumor miRNAs. Oncotarget.

[23] Ameri A, Ahmed HM, Pecho RDC, Arabnozari H, Sarabadani H, Esbati R (2023). Diverse activity of miR-150 in Tumor development: shedding light on the potential mechanisms. Cancer Cell Int.

[24] Hu T, Chong Y, Cai B, Liu Y, Lu S, Cowell JK (2019). DNA methyltransferase 1-mediated CpG methylation of the miR-150-5p promoter contributes to fibroblast growth factor receptor 1-driven leukemogenesis. J Biol Chem.

[25] Jiang X, Bugno J, Hu C, Yang Y, Herold T, Qi J (2016). Eradication of Acute Myeloid Leukemia with FLT3 Ligand-Targeted miR-150 Nanoparticles. Cancer Res.

[26] Li H, Ouyang R, Wang Z, Zhou W, Chen H, Jiang Y (2016). MiR-150 promotes cellular metastasis in non-small cell lung cancer by targeting FOXO4. Sci Rep.

[27] Jiang K, Shen M, Chen Y, Xu W (2018). miR-150 promotes the proliferation and migration of non-small cell lung cancer cells by regulating the SIRT2/JMJD2A signaling pathway. Oncol Rep.

[28] Tomicic MT, Dawood M, Efferth T (2021). Epigenetic Alterations Upstream and Downstream of p53 Signaling in Colorectal Carcinoma. Cancers.

[29] Zou SL, Chen YL, Ge ZZ, Qu YY, Cao Y, Kang ZX (2019). Downregulation of serum exosomal miR-150-5p is associated with poor prognosis in patients with colorectal cancer. Cancer Biomark Sect Dis Markers.

[30] Sun W, Zhang Z, Wang J, Shang R, Zhou L, Wang X (2016). MicroRNA-150 suppresses cell proliferation and metastasis in hepatocellular carcinoma by inhibiting the GAB1-ERK axis. Oncotarget.

[31] Yokobori T, Suzuki S, Tanaka N, Inose T, Sohda M, Sano A (2013). MiR-150 is associated with poor prognosis in esophageal squamous cell carcinoma via targeting the EMT inducer 1. Cancer Sci.

[32] Chen X, Xu X, Pan B, Zeng K, Xu M, Liu X (2018). miR-150-5p suppresses tumor progression by targeting VEGFA in colorectal cancer. Aging.

[33] Zhang Z, Wang J, Li J, Wang X, Song W (2018). MicroRNA-150 promotes cell proliferation, migration, and invasion of cervical cancer through targeting PDCD4. Biomed Pharmacother.

[34] Chen S, Wang Z, Dai X, Pan J, Ge J, Han X (2013). Re-expression of microRNA-150 induces EBV-positive Burkitt lymphoma differentiation by modulating c-Myb in vitro. Cancer Sci.

[35] Stamatopoulos B, Van Damme M, Crompot E, Dessars B, El Housni H, Mineur P (2015). Opposite Prognostic Significance of Cellular and Serum Circulating MicroRNA-150 in Patients with Chronic Lymphocytic Leukemia. Mol Med.

[36] Groot M, Lee H (2020). Sorting Mechanisms for MicroRNAs into Extracellular Vesicles and Their Associated Diseases. Cells.

[37] Kalluri R, McAndrews KM (2023). The Role of Extracellular Vesicles in Cancer. Cell.

[38] Ye R, Lin Q, Xiao W, Mao L, Zhang P, Zhou L (2023). miR-150-5p in neutrophil-derived extracellular vesicles associated with sepsis-induced cardiomyopathy in septic patients. Cell Death Discov.

[39] Zhou X, Zhao S, Li W, Ruan Y, Yuan R, Ning J (2021). Tubular cell-derived exosomal miR-150-5p contributes to renal fibrosis following unilateral ischemia-reperfusion injury by activating fibroblast in vitro and in vivo. Int J Biol Sci.

[40] Chang WA, Tsai MJ, Hung JY, Wu KL, Tsai YM, Huang YC (2021). miR-150-5p-Containing Extracellular Vesicles Are a New Immunoregulator That Favor the Progression of Lung Cancer in Hypoxic Microenvironments by Altering the Phenotype of NK Cells. Cancers.

[41] Zhou W, Yang F, Zhang X (2024). Roles of M1 Macrophages and Their Extracellular Vesicles in Cancer Therapy. Cells.

[42] Yugawa K, Yoshizumi T, Mano Y, Itoh S, Harada N, Ikegami T (2021). Cancer-associated fibroblasts promote hepatocellular carcinoma progression through downregulation of exosomal miR-150-3p. Eur J Surg Oncol.

[43] Sur D, Burz C, Sabarimurugan S, Irimie A (2020). Diagnostic and Prognostic Significance of MiR-150 in Colorectal Cancer: A Systematic Review and Meta-Analysis. J Pers Med.

[44] Cruz-Burgos M, Cortés-Ramírez SA, Losada-García A, Morales-Pacheco M, Martínez-Martínez E, Morales-Montor JG (2023). Unraveling the Role of EV-Derived miR-150-5p in Prostate Cancer Metastasis and Its Association with High-Grade Gleason Scores: Implications for Diagnosis. Cancers.

[45] Ozawa PMM, Vieira E, Lemos DS, Souza ILM, Zanata SM, Pankievicz VC (2020). Identification of miRNAs Enriched in Extracellular Vesicles Derived from Serum Samples of Breast Cancer Patients. Biomolecules.

[46] Scalavino V, Liso M, Serino G (2020). Role of microRNAs in the Regulation of Dendritic Cell Generation and Function. Int J Mol Sci.

[47] Xu SJ, Hu HT, Li HL, Chang S (2019). The Role of miRNAs in Immune Cell Development, Immune Cell Activation, and Tumor Immunity: With a Focus on Macrophages and Natural Killer Cells. Cells.

[48] Smith NL, Wissink EM, Grimson A, Rudd BD (2015). miR-150 Regulates Differentiation and Cytolytic Effector Function in CD8+ T cells. Sci Rep.

[49] Hirayama D, Iida T, Nakase H (2017). The Phagocytic Function of Macrophage-Enforcing Innate Immunity and Tissue Homeostasis. Int J Mol Sci.

[50] Liu Y, Zhao L, Li D, Yin Y, Zhang CY, Li J (2013). Microvesicle-delivery miR-150 promotes tumorigenesis by up-regulating VEGF, and the neutralization of miR-150 attenuate tumor development. Protein Cell.

[51] Cao Y, Cao D, Zhu T (2024). Circular RNA FMN2 motivates colorectal cancer development by mediating tumor-associated macrophage polarization by controlling the microRNA-150-5p/PIK3R3 axis. Electron J Biotechnol.

[52] Zhu J, Yao K, Guo J, Shi H, Ma L, Wang Q (2017). miR-181a and miR-150 regulate dendritic cell immune inflammatory responses and cardiomyocyte apoptosis via targeting JAK1-STAT1/c-Fos pathway. J Cell Mol Med.

[53] Pesce S, Greppi M, Ferretti E, Obino V, Carlomagno S, Rutigliani M (2020). miRNAs in NK Cell-Based Immune Responses and Cancer Immunotherapy. Front Cell Dev Biol [Internet]. 2020 Feb 25 [cited 2025 May 21];8. Available from: https://www.frontiersin.org/journals/cell-and-developmental-biolog/articles/10.3389/fcell.

[54] LeBien TW, Tedder TF (2008). B lymphocytes: how they develop and function. Blood.

[55] Loder BF, Mutschler B, Ray RJ, Paige CJ, Sideras P, Torres R (1999). B Cell Development in the Spleen Takes Place in Discrete Steps and Is Determined by the Quality of B Cell Receptor-Derived Signals. J Exp Med.

[56] Cano RLE, Lopera HDE (2025). Introduction to T and B lymphocytes. In: Autoimmunity: From Bench to Bedside [Internet] [Internet]. El Rosario University Press; 2013 [cited.

[57] Zhou B, Wang S, Mayr C, Bartel DP, Lodish HF (2007). miR-150, a microRNA expressed in mature B and T cells, blocks early B cell development when expressed prematurely. Proc Natl Acad Sci.

[58] Thomas MD, Kremer CS, Ravichandran KS, Rajewsky K, Bender TP (2005). c-Myb is critical for B cell development and maintenance of follicular B cells. Immunity.

[59] Jiang XX, Liu Y, Li H, Gao Y, Mu R, Guo J (2016). MYSM1/miR-150/FLT3 inhibits B1a cell proliferation. Oncotarget.

[60] Kluiver JL, Chen CZ (2012). MicroRNAs regulate B-cell receptor signaling-induced apoptosis. Genes Immun.

[61] Muljo SA, Ansel KM, Kanellopoulou C, Livingston DM, Rao A, Rajewsky K (2005). Aberrant T cell differentiation in the absence of Dicer. J Exp Med.

[62] Zhang N, Bevan MJ (2010). Dicer controls CD8+ T-cell activation, migration, and survival. Proc Natl Acad Sci.

[63] Xia S, Huang J, Yan L, Han J, Zhang W, Shao H (2022). miR-150 promotes progressive T cell differentiation via inhibiting FOXP1 and RC3H1. Hum Immunol.

[64] Ban YH, Oh SC, Seo SH, Kim SM, Choi IP, Greenberg PD (2017). miR-150-Mediated Foxo1 Regulation Programs CD8+ T Cell Differentiation. Cell Rep.

[65] Chen Z, Stelekati E, Kurachi M, Yu S, Cai Z, Manne S (2017). miR-150 Regulates Memory CD8 T Cell Differentiation via c-Myb. Cell Rep.

[66] Ménoret A, Agliano F, Karginov TA, Karlinsey KS, Zhou B, Vella AT (2023). Antigen-specific downregulation of miR-150 in CD4 T cells promotes cell survival. Front Immunol [Internet]. 2023 Jan 27 [cited 2025 May 26];14. Available from: https://www.frontiersin.org/journals/immunology/articles/10.3389/fimmu.

[67] Huemer F, Leisch M, Geisberger R, Zaborsky N, Greil R (2021). miRNA-Based Therapeutics in the Era of Immune-Checkpoint Inhibitors. Pharmaceuticals.

[68] Bajan S, Hutvagner G (2020). RNA-Based Therapeutics: From Antisense Oligonucleotides to miRNAs. Cells.

[69] Neumeier J, Meister G (2020). siRNA Specificity: RNAi Mechanisms and Strategies to Reduce Off-Target Effects. Front Plant Sci [Internet]. 2021 Jan 28 [cited 2025 May 26];11. Available from: https://www.frontiersin.org/journals/plant-science/articles/10.3389/fpls.

